# Mixed Papilloma of the Lacrimal Sac as a Cause of Hemolacria

**DOI:** 10.1155/crot/6290800

**Published:** 2026-05-22

**Authors:** Carly Malesky, Madina Falcone, Poornima Hegde, Todd E. Falcone

**Affiliations:** ^1^ School of Medicine, University of Connecticut, Farmington, Connecticut, USA, uconn.edu; ^2^ Division of Ophthalmology, UConn Health Center, Farmington, Connecticut, USA; ^3^ Department of Pathology and Laboratory Medicine, UConn Health Center, Farmington, Connecticut, USA, lhsc.on.ca; ^4^ Division of Otolaryngology—Head and Neck Surgery, UConn Health Center, Farmington, Connecticut, USA

## Abstract

Inverted papillomas (IPs) derived from the lacrimal sac are a rare pathology, with only seven cases reported in the literature.^1,2 “^Mixed” endophytic and exophytic papillomas of the lacrimal sac are even more rare, with no reports in the literature. We present the case of a 55‐year‐old man who presented to the clinic with unilateral hemolacria. Multiple imaging modalities revealed a mass in the lacrimal sac, which was subsequently surgically excised by an otolaryngologist and oculoplastic surgeon. Pathologic analysis of the specimen demonstrated both exophytic and endophytic growth patterns, suggestive of a mixed papilloma subtype. This case highlights the importance of prompt, accurate diagnosis and interdisciplinary management, as patients require regular clinical follow‐up to monitor recurrence and malignant transformation.

## 1. Case Presentation

**FIGURE 1:**
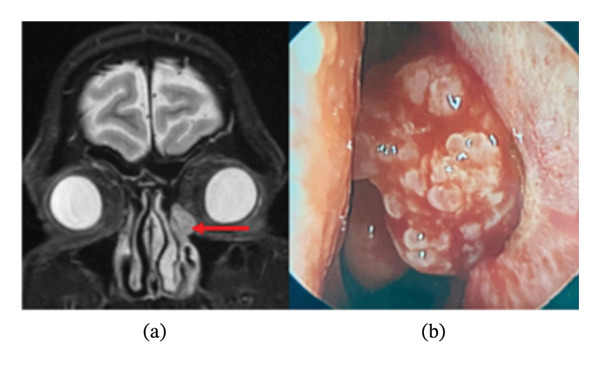
(a) Coronal T2‐weighted MRI of the orbits with IV contrast demonstrating a 1.1 × 1.1 × 1.5 cm enhancing lesion in the left lacrimal sac, with no evidence of bony erosion (red arrow). (b) Intraoperative nasal endoscopy of the mass following incision of the lacrimal sac.

A 55‐year‐old male with a history of allergic rhinitis presented to his primary care provider with 2 months of transient left‐sided hemolacria and chronic epiphora. His symptoms progressed to include an intermittent pulsing sensation in the left eye, but he denied pain, diplopia, blurry vision, anosmia, nasal congestion, or epistaxis. Magnetic resonance imaging (MRI) of the orbits with contrast demonstrated a 1.1 × 1.1 × 1.5 cm enhancing lesion in the left nasolacrimal duct, indistinct from the lacrimal sac (Figure 1(a)). Upon presentation to otorhinolaryngology clinic, the mass was not visible or palpable externally and nasal endoscopy revealed normal mucosa on the lateral wall. Oculoplastic evaluation revealed left hemolacria and nasolacrimal duct obstruction. Computed tomography (CT) imaging revealed a 1 × 1.2 × 1.4 cm well‐defined and homogeneous soft tissue mass within the left lacrimal sac, with no evidence of bony erosion. The patient subsequently underwent left endoscopic dacryocystorhinostomy (DCR). The left lacrimal sac demonstrated significant papillomatous tumor burden in continuity with the lacrimal sac mucosa, and additional dacryocystectomy was performed (Figure 1(b)). Given the size of the tumor, an external tear trough incision was made to facilitate complete lacrimal sac removal.

## 2. Discussion

**FIGURE 2:**
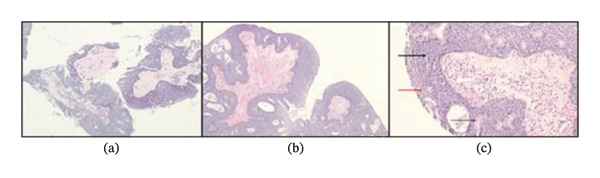
(a–c) Histologic examination utilizing routine hematoxylin and eosin staining. (a) Low power examination (40 × magnification) reveals detached finger‐like projections representing branched fibrovascular cores lined by multilayered epithelium consistent with an exophytic growth pattern. (b) Higher power examination (100 × magnification) reveals broad papillary structures covered by multilayered epithelium including cystic spaces and occasional intraepithelial glandular structures lined by goblet cells. (c) At 400 × magnification, transitional epithelium appears multilayered (thick black arrow) with focal squamous differentiation (red arrow); intraepithelial glandular structures lined by goblet cells (thin black arrow) and cystic spaces are noted.

Sinonasal papillomas, or Schneiderian papillomas, are benign epithelial neoplasms derived from respiratory epithelium and account for 0.5%–4% of all sinonasal neoplasms [1, 2]. Histological subtypes of Schneiderian papillomas include inverted, exophytic, and oncocytic papillomas [2]. Specimens containing more than one of these subtypes are classified as mixed papillomas [3]. Previous studies have reported that up to 4.7% of sinonasal papillomas exhibit both inverted and exophytic subtypes [3]. The pathological examination of this lacrimal sac specimen revealed multilayered transitional epithelium with an intact basement membrane, featuring an exophytic lesion with a fungiform growth pattern and a small endophytic component (Figures 2(a), 2(b), and 2(c)). Based on our pathologist’s review, these findings could represent a mixed papilloma. However, this is somewhat controversial as inverted papillomas can have areas that may appear exophytic.

Tumor debulking is generally not recommended due to high recurrence rates of inverted papillomas in both the sinonasal cavity and lacrimal sac. All previous reports of mixed sinonasal papillomas in the literature are localized to the nasal cavity, most frequently the nasal septum, and had a 100% local recurrence rate [3]. In contrast, patients studied with inverted papillomas of the lacrimal sac demonstrated a recurrence rate of 14%, with no cases of malignant transformation observed [4]. One case report describes an exophytic papilloma of the lacrimal sac with incomplete resection at the bony canal duct, leading to recurrence and extension into the nasal cavity through the nasolacrimal duct [5]. Malignant transformation has been reported in 5%–15% of inverted papillomas of the nasal cavity [5, 6].

The pathophysiology of Schneiderian papillomas and their subtypes is not yet fully understood. Low‐risk HPV subtypes have been identified in a significant proportion of exophytic and inverted papillomas in the sinonasal cavity [7]. Conversely, high‐risk HPV subtypes are more frequently associated with inverted papillomas that have undergone malignant transformation [7].

The differential diagnosis of a lacrimal sac mass includes benign epithelial tumors such as papillomas, oncocytomas, and adenomas [8]. Malignant epithelial tumors of the lacrimal sac, such as squamous cell carcinoma, transitional cell carcinoma, and oncocytic adenocarcinoma are rare [8]. Nonepithelial tumors, which are more rare, encompass mesenchymal, melanocytic, and lymphoproliferative tumors [8]. The most common presenting symptoms of patients with lacrimal sac tumors are clear or bloody epiphora, painless swelling localized to the medial canthus, and nasal obstruction [8].

Lacrimal sac tumors are difficult to differentiate radiographically. However, CT imaging proves valuable in assessing the extent of bone erosion and potential local invasion, which would support the presence of a malignant process [7]. MRI is indicated in patients for which there is concern for an invasive or malignant process or if CT findings suggest soft tissue extension [7]. Definitive diagnosis of lacrimal sac tumors requires incisional or excisional biopsy [9]. Fine needle aspiration is not recommended due to the risk of hemorrhage and inadequate sampling [9].

Both an endoscopic and external operative approach performed by a skilled otolaryngologist and oculoplastic surgeon are imperative to reduce tumor burden, risk of recurrence, and malignant transformation. Endoscopic DCR is considered to be the standard of care for addressing pathologies of the lacrimal system distal to the canaliculi [1]. This technique offers cosmetic advantages. However, it is not appropriate as a standalone intervention for large, invasive, or advanced‐stage tumors or in patients with significantly distorted anatomy. DCR is appropriate for benign tumors confined to the lacrimal sac confirmed with intraoperative frozen section analysis. In cases where disease extends beyond the sac, more extensive surgical approaches may be required to achieve adequate oncologic margins of 1–1.5 cm [10].

Our patient is currently 20 months out from surgery with no signs of recurrence. Prompt and accurate diagnosis is essential to monitor recurrence and malignant transformation. Postoperative surveillance following DCR is recommended every 3 months during the first year to assess rhinostomy patency and identify any mucosal changes concerning for malignant transformation that would require biopsy [11]. From one to five years postoperatively, nasal endoscopy may be performed every 3–6 months, with annual examinations thereafter [11]. Repeat imaging is recommended in patients with persistent or recurrent symptoms, even following a normal postoperative endoscopy, especially when the initial endoscopy was normal.

## Funding

The authors received no financial support for the research, authorship, and/or publication of this article.

## Ethics Statement

Our institution does not require ethics approval for reporting individual cases or case series.

## Consent

No written consent has been obtained from the patients as there is no patient identifiable data included in this case report/series.

## Conflicts of Interest

The authors declare no conflicts of interest.

## Data Availability

Data availability is not applicable to this article as no new data were created or analyzed in this study.
